# Addressing inequities in the design and delivery of prehabilitation in the UK: case studies of challenges, complexities and good practice^*^

**DOI:** 10.1111/anae.70286

**Published:** 2026-07-20

**Authors:** Charlotte Hadley, Xiubin Zhang, Hilary Stewart, Sophie Stanley, Ces Kulikowski, Andrea Partridge, Laura Wareing, Lisa Ashmore, Christopher Gaffney, Jo Rycroft Malone, Andrew Smith, Cliff Shelton

**Affiliations:** ^1^ Lancaster Medical School Lancaster University Lancaster UK; ^2^ North West School of Anaesthesia Health Education England Manchester UK; ^3^ Patient Co‐Investigator Lancaster UK; ^4^ Faculty of Health and Medicine Lancaster University Lancaster UK; ^5^ Department of Anaesthesia University Hospitals Morecambe Bay NHS Foundation Trust Lancaster UK; ^6^ Department of Anaesthesia Wythenshawe Hospital Manchester UK

**Keywords:** health inequities, nutrition therapy, prehabilitation, pre‐operative exercise, psychology

## Abstract

**Introduction:**

Prehabilitation has become increasingly prominent in the context of cancer surgery. While prehabilitation offers significant benefits, it can also inadvertently broaden inequalities because it is often more accessible to some than others. To explore the connection between health inequalities and prehabilitation before cancer surgery, we designed a qualitative study based on case study methodology.

**Methods:**

Diverse prehabilitation services were purposively sampled. We conducted orientation interviews with service leads; semi‐structured interviews with professionals, patients and carers; and observations of prehabilitation practice. These were conducted following topic guides informed by implementation science theory. We employed an inductive thematic approach to allow themes to emerge, highlighting connections between prehabilitation and health inequalities.

**Results:**

Six prehabilitation services across England and Wales were included, comprising a combination of established and new services, urban and semi‐rural settings and varied healthcare delivery models. We conducted 47 interviews with professionals, 47 interviews with patients (including eight with carers) and 28 observations of practice. We developed six themes, which illuminated how: patients' circumstances influence their ability to engage; the value of prehabilitation to patients depends on how it is introduced and understood; services can address, but also exacerbate, health inequalities; services may offer more and/or less than the prototypical prehabilitation model; services use and develop an incomplete evidence base; and services work with limited resources to provide the most benefit.

**Discussion:**

Our findings highlight that while prehabilitation is often presented as a simple concept in the literature, in practice it is a complex intervention that must integrate with other ongoing treatments (e.g. surgery, chemotherapy, radiotherapy) during an already challenging period in patients' lives. Our qualitative findings from real‐world settings illustrate how and why disparities arise in practice and offer insights for more equitable prehabilitation.

## Plain Language Summary

**What we did:**

We studied six services in England and Wales that help people get ready for cancer surgery. These programmes, called prehabilitation services (often called ‘prehab’), support patients before treatment. We interviewed healthcare workers, patients and carers, and we also watched how the services worked in practice.

**Why did we do it:**

Not everyone has the same chance to use prehabilitation services. People's health, where they live, and their personal circumstances can affect whether they can take part. We wanted to understand why these differences happen and how services could be made fairer for everyone.

**What we found:**

We found that prehabilitation is not the same for every patient. Some people found it easier to access and benefit from the support than others. Services often had to adapt to meet different patients' needs, and many work with limited staff and resources. We also found that cancer treatment can be a difficult and stressful time, making it harder for some people to take part. The study showed that more work is needed to make prehabilitation services fair and accessible for all patients.

## Introduction

Prehabilitation, typically comprising interventions designed to improve patients' physical, nutritional and/or psychological readiness before major treatments, has gained traction in recent years. Prehabilitation is associated with improved health outcomes, fewer postoperative complications and enhanced recovery [1, 2], with evidence in several settings, particularly cancer surgery [2, 3, 4, 5, 6]. Nonetheless, the configuration of prehabilitation services across the UK remains highly variable [7, 8, 9], which risks exacerbating existing health inequities, particularly for underserved groups [10, 11, 12, 13]. Most research to date has focused on the impact of participation in prehabilitation, with less attention to the factors associated with non‐participation. Consequently, there is little understanding of how these inequities emerge in practice or how services mitigate these disparities [14, 15]. Without understanding these factors, prehabilitation services lack a robust basis for addressing health inequalities.

The design and implementation of prehabilitation services can place varying demands on patients' time, energy and resources. It may require access to transportation, equipment and appropriate nutrition. Patients also require the capacity, knowledge, skills and financial means to engage. Access to these resources and amenities is distributed unevenly across the UK [14], which may create inequities in participation. In the context of cancer surgery, these challenges may be further shaped by patients' experiences of their illness and how it is managed.

Equitable access to prehabilitation is important not only for improving individual patient care but also for supporting broader health equity goals [16]. To better understand this context, we designed a mixed‐methods project – Prehabilitation Before Cancer Surgery: Quality and Inequality (PARITY) – to examine how inequities (unfair, avoidable differences in what patients experience) arise and how inequalities (differences in the distribution of resources) are addressed within NHS prehabilitation services. This incorporated three sub‐studies: a Delphi study on factors influencing quality and equity in prehabilitation [17]; a service mapping study to identify prehabilitation services, their content, funding model, and delivery [7]; and case studies of prehabilitation services. The aim of this study was to examine case studies representing the range of prehabilitation service models identified in our mapping study [7] to identify examples of good practice, including efforts to address inequalities and to explore the contextual factors that influence service implementation.

## Methods

Ethical approval for the study was granted by the Bradford Leeds NHS Research Ethics Committee. We employed a case study approach [18] underpinned by an interpretivist perspective [19], combining in‐depth interviews with health and prehabilitation professionals, patients and relatives/carers, along with observations of practice, across six prehabilitation services. This enabled us to develop a rich understanding of practice in situ as well as the experiences of those who design and deliver services, and those who engage with them.

Each case study site was purposively selected based on data from our earlier mapping study [7], which provided a snapshot of services across the UK. We identified services that exemplified differences in terms of funding sources, geographic location, service duration, design and delivery, enabling us to think critically about the mechanisms that facilitate, discourage or prevent initial engagement and sustained participation in prehabilitation. The proposed research sites were reviewed and agreed upon in collaboration with a patient and public advisory panel.

Drawing on previous research [20, 21], we planned initially to conduct approximately ten interviews with professionals; ten with patients (and relatives/carers); and five to eight observations at each site. However, the final sample size and the decision to close research sites were guided by the principles of information power [22] and influenced by practical considerations (e.g. the needs of the prehabilitation service). Our protocol proposed eight research sites across the UK, including at least one in Wales, Scotland and Northern Ireland [23]. However, we were unable to recruit sites in Northern Ireland as those sites had existing commitments to other research, and we were unable to identify eligible services in Scotland [7]. We therefore narrowed the scope of the study to six England and Wales‐based sites, in agreement with the study oversight group and the funder.

Written informed consent was provided by participants except in the single case where a participant was lacking mental capacity [24], when the declaration of a consultee was sought. For observations conducted in public or large‐group settings where obtaining individual consent was not feasible, posters or virtual backgrounds were displayed to indicate that observations were taking place and provide instructions on how to opt out. We began data collection by conducting a semi‐structured ‘orientation’ interview with prehabilitation service lead(s) (online Supporting Information Appendix S1). This was designed to supplement our existing data [7] and provide a detailed understanding of the service (e.g. about funding, how the service is managed and where prehabilitation takes place).

A local collaborator (usually the service lead) acted as a ‘gatekeeper’ and facilitated access to the service and potential participants. In most cases, local collaborators worked closely with our team to help identify and recruit potential participants and arrange site visits. This was not always straightforward. In one instance, access was delayed due to setbacks in the setup of the prehabilitation service. In another, the service lead temporarily withheld access due to a critical incident at the hospital. Consequently, the research team spent more time ‘in the field’ at some research sites than others. At some research sites, the service lead provided a weekly schedule of prehabilitation activities. These schedules became useful methodological tools that enabled us to observe a diverse range of prehabilitation activities during our site visits.

We sought involvement from patients awaiting cancer surgery who had experienced prehabilitation, and those who had not (typically because their cancer type was not eligible for prehabilitation in their institution). Participants were recruited through one of two approaches: some were identified and approached by professionals who acted as ‘gatekeepers’; others were recruited via convenience sampling if they had an appointment with the prehabilitation or pre‐operative team on days when the research team was present.

All interviews and observations were informed by topic guides, drawing on the Promoting Action on Research Implementation in Health Services (PARiHS) framework, and developed with input from our patient and public involvement group and trialled with volunteers from within the research team (online Supporting Information Appendices S2 and S3) [25, 26]. Applying this framework across two data collection methods allowed us to examine phenomena from complementary perspectives, capturing work as articulated in the interviews and work as done in practice. This framework also aligned with our focus on implementation, as well as insights from earlier stages of the project [7, 17]. Interviews were conducted in person, via videoconferencing (Microsoft Teams, Microsoft Inc., Redmond, WA, USA) or by telephone, according to the participant's preference. Observations were recorded as shorthand fieldnotes. These were then used as prompts to help us reflect upon events and create more detailed fieldnotes at the earliest opportunity. Documentary sources of information (e.g. patient leaflets, posters) were also used to help develop our understanding of each prehabilitation service. To minimise participant burden, interviews were conducted flexibly and the interview schedule was adapted and shortened when participants had limited time.

All interviews were audio‐recorded and transcribed verbatim by a professional transcriber under a confidentiality agreement. All interview transcripts and fieldnotes were pseudonymised by allocation of institutional and individual pseudonyms, and the removal of geographical and personal details to maintain the confidentiality of participants and the research site. Data collection and analysis was led by a team of co‐authors (CH, HS, XZ, SS and CS). The research team regularly reflected on the insights being gathered, and how their own experiences and positionality impacted the data being collected and its interpretation. These discussions considered how our professional backgrounds and experience (CH, a social scientist; HS, a healthcare sociologist; XZ, a health services researcher with a nursing background; and SS and CS, academic anaesthetists) and our personal and professional experiences of cancer treatment and broader life circumstances (e.g. work‐life balance, financial (in)security) could shape the data we were collecting and analysing.

We undertook an inductive thematic analysis drawing on Braun and Clarke's method (i.e. familiarisation; initial coding; searching for themes; reviewing themes; defining and naming themes; producing the report) [27]. Analysis was facilitated by cloud‐based qualitative data management software (Quirkos Cloud, Quirkos, Edinburgh, UK), which enabled team members to code simultaneously. We developed a shared codebook and met frequently to discuss codes, promoting consistency across the analysis [28]. While defining themes, we conducted a workshop with co‐investigator AP (a patient advocate with lived experience of cancer treatment) to examine how the themes resonated with her own experiences. Themes were subsequently refined following this workshop and during manuscript preparation. This report is prepared in accordance with the Standards for Reporting Qualitative Research [29].

## Results

Six prehabilitation services across England and Wales were recruited (Table 1). These services varied in their service duration, geographical settings and approach to service delivery. Physical fitness, nutritional and psychological‐based interventions were delivered to differing extents and through a variety of formats, from one‐to‐one in‐person sessions to online group classes. Data collection was conducted between August 2024 and March 2025.

**Table 1:** Characteristics of included services.

Site*/region	Catchment	Service types	NHS Trust size**	Care setting	Delivery mode(s)	Years since establishment
Wansfell, North England	Urban	Specialist/acute‐general	Large	Hospital	Face‐to‐face	> 5 years
Rosthwaite, North England	Urban	Acute‐general/specialist/community	Large	Community	Face‐to‐face	> 5 years
Greystones, Wales	Semi‐rural	Acute‐general/community	Large	Hospital, community	Face‐to‐face, telephone, videoconferencing	< 5 years
Dalehead, North England	Semi‐rural	Acute‐general/community	Medium	Hospital, community	Face‐to‐face, telephone	< 5 years
Newlands, South England	Urban	Acute‐general/specialist	Large	Virtual	Videoconferencing, telephone	< 5 years
Robinson, South England	Urban	Acute‐general/specialist	Medium	Hospital	Face‐to‐face	In active set‐up

* Site names are pseudonyms.

** Based on staff numbers: small < 3000; medium 3000–9000; large > 9000.

In total, 50 healthcare professionals, 61 patients and 11 relatives/carers participated in the study. Demographic details are provided in online Supporting Information Table S1. This includes those who participated in interviews and small‐group observations, but not those who participated in public/large‐group activities where individual consent was not obtained.

A total of 94 interviews were conducted: 47 with healthcare professionals and 47 with patients. Eight patient interviews also included relatives/carers, with a total of 11 participating. The median (IQR [range]) duration of healthcare professional interviews was 46 (27 [22–75]) min and of patient interviews was 30 (16 [11–92]) min. Patients reported varying levels of engagement with prehabilitation, ranging from no engagement to weekly participation.

Access to patient participants was, in part, influenced by gatekeeping practices within prehabilitation teams, sometimes through identifying ‘good’ or ‘potential’ cases. This may have shaped the sample to favour participants who were more engaged, with more complex or uncertain cases being underrepresented. This emphasises the importance of including patients who had not received prehabilitation.

We conducted 28 observations of practice across the six sites (Table 2). All observations were overt (i.e. the participants were aware that they were being observed) and involved a varying degree of participation (e.g. on occasion, researchers were invited to engage in conversation or to join in with exercise classes) [28].

**Table 2:** Characteristics of observations (n = 28). Values are number (proportion).

Observation type	Setting(s)	Total
Initial assessments	Hospital, patients' homes, leisure centres	12
Review assessments	Hospital, patients' homes, leisure centres, telephone	7
Gym induction sessions	Leisure centres, hospital	3
Exercise classes	Online, leisure centres	4
Multidisciplinary team meetings	Online	1
Social exercise sessions	Local community	1

Our observational data provide insights into day‐to‐day practice, patient‐healthcare professional dynamics and the contextual factors shaping prehabilitation delivery. We observed firsthand how prehabilitation services operate amid the everyday challenges of communication, workload and limited resources. These elements were not always visible through interviews alone.

We identified six themes relating to the equitable implementation of prehabilitation services (online Supporting Information Figure S1). The quotes presented below reflect accounts that provide detailed insight into the themes.

### Theme 1 – How patients' circumstances influence their ability to engage

Patient engagement is recognised as being crucial to the success of prehabilitation [30]. Patients must introduce and integrate new activities (e.g. regular aerobic exercise) and behaviour changes (e.g. dietary modification) into their daily routines, placing additional demands on their time and energy. Our data show that, particularly in the early stages after diagnosis, patients are often simultaneously processing both their diagnosis and information from their healthcare team about treatment options. During this period, while awaiting further communication from their healthcare team, patients describe their experience as emotionally unsettling, marked by apprehension, uncertainty, sadness, frustration and a sense of being overwhelmed. In addition, cancer treatment and its associated symptoms create additional challenges to engagement with prehabilitation. The extract below provides insights into early experiences following diagnosis:

*Interviewer:*
*‘[How] did you feel after you were diagnosed?’*

*Noah:*
*‘Well, it's … it's funny now, really, it's settling in now, considering obviously’*.

*Noah's wife:*
*‘Such a shock.’*

*Noah: ‘*
*Just got to wait now to see what they can do for me. This is the thing… I was waiting to know what they can do for me, if they can do anything, if I can have an operation, or I can't, I don't know.’*

*[After his initial reaction, he goes on to explain how he feels now]*.

*Noah:*
*‘Well, you more or less go into denial, you try to forget it, put it to the back of your mind, you know? It raises itself all the time, especially at night, when you wake up in the night and you're thinking about it.’*

Noah, Patient (experience of prehabilitation), Interview, Greystones

For many patients, learning about their diagnosis and treatment prompted them to engage in practical or spiritual forms of preparation, such as ‘decluttering the home’; practicing mindfulness; and getting their personal affairs in order. While awaiting surgery, these activities helped patients address practical concerns and momentarily divert their attention. Consequently, they became meaningful ways in which patients make use of the time leading up to surgery. However, performing these activities also takes up time, leaving less opportunity to engage in other activities:

‘… I've got to show somebody how to run my business in case things go really badly. I have [to] write my will, I have to get powers of attorney done. Yeah, I've got till [date] when my surgery is. So that's the other thing, because if I was just to sit at home, just doing nothing, I would fret, I would be unhappy, so all I'm going to do, I'm just going to bloody distract myself by working bloody hard while I can, for three weeks before somebody rips a hole in my stomach and takes out all my organs…’

Frankie, Patient (experience of prehabilitation), Interview, Dalehead

As noted above, participation relies on the time, energy and other resources that patients can commit while managing other competing priorities. This relates not only to the emotional and cognitive demands of preparing for treatment but also to the ongoing demands of their everyday lives. Many participants described managing pre‐existing health conditions, impairments or disabilities and coping with symptoms. In addition, many participants balanced caring responsibilities and other commitments, leaving little or no time for themselves:

*Penelope:*
*Discussed current treatment (hormone tablets) – patient experiencing bone pain side effect. Discussed asthma – taking steroids for 5–6 years; caused diabetes; not able to have surgery yet as diabetes not well controlled enough*.

*Occupational Therapist:*
*‘How are you feeling in yourself in terms of everything going on?’*

*Penelope:*
*Talked about caring responsibilities for adult son (who is living with severe disability because of cerebral palsy). Gets help with caring for son from two carers per day. Discussed caring arrangements for son after surgery – plan for second adult son to be paid for this. Sleeps in same room as son – currently poor sleep. Plan discussed to support sleep after surgery*.

*Occupational Therapist:*
*‘What's your support network?’*

*Penelope:*
*Mentioned her youngest son and carer who is close friend*.

*Occupational Therapist:*
*‘It's completely understandable… that when you get a date [for surgery] the nerves and the worries get worse.’ Enquiry about relaxation techniques*.

*Penelope:*
*‘What's relaxation?!’*

Observation notes taken during a patient's initial assessment, Wansfell

During this consultation, Penelope articulates the strain of managing her own needs while being a full‐time carer, showing how the demands of caregiving can significantly limit autonomy. Beyond this, many patients rely on others for care and/or transportation to hospital appointments, but often these carers/relatives have their own conflicting demands. The excerpt below illustrates how the reliance on family support and other (indirect) constraints can influence the capacity of a patient to engage with prehabilitation:

*Physiotherapist:*
*‘The other part of what we do is classes…you're in the middle of [Anita's hometown] …’. He indicates that the nearest locations where these classes are available are at [Leisure Centre 4] and [Hospital 19]*.

*Anita's son:*
*Explains that – ‘transport is an issue’. Anita's son drove Anita to her appointment today but took time off work to do so. He then questioned the benefit of attending the classes, due to the timing – ‘Christmas isn't too far away and if she gets a date in Jan…’*.

*Physiotherapist:*
*Reassured Anita's son, explaining that there are benefits of performing the exercises for at least 7–10 days before surgery*.

*Anita's son:*
*Replies, ‘ah okay’*.

*Physiotherapist:*
*Asks Anita – ‘do you think you can commit today?’*

*Anita's son:*
*Responds – ‘We just need to figure out the logistics.’*

Observation notes taken during Anita's initial prehabilitation assessment, Greystones

Although the physiotherapist attempts to engage with Anita (the patient) in the exchange above, the negotiation takes place with her son, illustrating how the commitments of carers/relatives can influence participation by virtue of their role in aiding with activities of daily living. Symptoms, impairments and commitments are important in this context because prehabilitation requires active participation. Taken together, these examples illustrate that, beyond motivation and emotional resilience, there are broader practical, logistical and social influences that shape whether patients can engage. This underscores the importance of designing and implementing prehabilitation services in ways that minimise additional burden during an already difficult period.

### Theme 2 – How the value of prehabilitation to patients depends on how it is introduced and understood

Most patients recognise the potential value of prehabilitation, yet many were acutely aware that this did not necessarily translate into clear benefits for them. This was influenced by preconceptions about upcoming healthcare interventions; personal and healthcare circumstances; perceived gaps in care; and the timing of interventions within the care pathway. Patients generally perceived prehabilitation to be valuable because of its potential to shape their postoperative health outcomes and maintain a sense of control during the waiting period, resonating with the meaningful activities described above (Theme 1). Patients often made strong connections between physical fitness and dietary optimisation, and reducing peri‐operative risk:

*Interviewer:*
*‘So in terms of you coming here for prehabilitation, then, how did you decide that you did want to do it? Was it a shared decision between you and‐’*

*Kevin:*
*‘I want to live, I want to be as fit as possible going into the operation, that's it.’*

Kevin, Patient (experience of prehabilitation), Interview, Rosthwaite

Above, Kevin draws a clear association between being ‘fit’ and a reduced risk of peri‐operative mortality. Patients also connected nutrition and risk:

*‘I was always going to try to do it myself, but it's easy to slip and fall off. So where you're phoning them regular, or they're phoning me regularly, to be able to say, “I've gone from 16 [stone] to 15,” “I've gone from 15 to 14 and a half, from 14 and a half to 14,” it's encouraging that you can keep saying, because you don't want to say, “This month, I'm 14,” and phone them back in six weeks, “I'm now 15 stone 2”. So, where you're encouraged, because you're talking to someone, you're reporting regularly on your physical being…’*

Jake, Patient, (experience of prehabilitation), Interview, Newlands

Here, Jake describes his progress in terms of weight loss, enabled by the continuous support of prehabilitation professionals. While this concept of nutritional wellness aligns more closely with societal ideals of body image [31] than the nutritional goals of peri‐operative care (which typically advocate against intentional weight loss [32, 33]), it represents a positive impact from Jake's perspective. In these instances, prehabilitation serves as a catalyst through which patients can support themselves and reduce the risk of complications. Consequently, the timing of prehabilitation interventions is critical, determining the degree to which patients can make use of this opportunity before surgery.

Despite the value of prehabilitation to many patients, some perceived prehabilitation as a missed opportunity, particularly when the intervention was seen as irrelevant (e.g. exercise interventions offered to patients who already exercise recreationally) or when it was offered at a late stage:

*‘[Prehabilitation is] rubbish, because‐ simply because‐not a lot has been happening with me and I gather that I should have probably been doing all this now a few weeks back, rather than‐ but it was only decided the operation was going to go ahead last week, you know, so I can't really say that – I've not benefited from it, because there's so little time in between the [decision] and the operation, you know?’*

James, Patient (experience of prehabilitation), Interview, Greystones

Above, James, who attended his first prehabilitation appointment less than a week before surgery, expresses his dismay, realising that he “*should have probably been doing all this now a few weeks back”*. Taken together, these examples illustrate how the success of prehabilitation partly depends on its integration and coordination with other aspects of treatment and care.

While prehabilitation is offered to an increasing number of patients nationwide, there are many for whom it is not available, either because no service exists where they are receiving treatment [7] or because they are ineligible for inclusion in a service. Some patients identified ways in which prehabilitation could have been useful in their care. For example, Cynthia (below) turned to the internet for information regarding the role of diet:

***‘**I think [prehabilitation would be] useful because I've asked a few questions about nutrition, things like, you know, “Should I be drinking soya milk,” because you read about soy containing oestrogen, and I think sometimes there's this slight gap, because I can look at menopausal things and I can look at breast cancer things, but there's not many that combine the two, so when I came off [hormone replacement therapy], I was like Googling… So, there's a few little gaps in there, and I think that would have been perhaps quite useful to be able to find a bit more about that, have some about that.’*

Cynthia, Patient (no experience of prehabilitation), Interview, Robinson

Although the restrictive inclusion criteria of many prehabilitation services is perhaps the most explicit type of “*intervention‐generated inequality*” in our data [34], even amongst patients for whom prehabilitation is technically available, other systemic and structural inequities in access exist.

### Theme 3 – How services can address, but also exacerbate, health inequalities

It is well‐recognised that some groups (e.g. those living in socio‐economic deprivation, certain ethnic minority groups, patients living with frailty and/or multimorbidity) face greater barriers to engaging with prehabilitation [10, 11, 12, 13]. Patients who fall within these groups also face higher peri‐operative risks [35, 36]. Therefore, prehabilitation could be less accessible for those who would benefit the most.

Staff at all case study sites were aware of the implications of inequitable access to prehabilitation and had implemented measures to mitigate against this. These strategies varied between sites, shaped by the resources available (e.g. space, finances) and the perceived needs of the population served:

*‘… we have a really poor population in [our city], and exercise doesn't feature in terms of their day‐to‐day living, I suppose, and we also have, therefore, a poor population in terms of their fitness to undergo treatment. So even though we've got lots of treatment, people don't get treatment because their fitness is so poor that they can't tolerate it… there's a two‐pronged attack… at the moment, especially in relation to lung cancer, that we need to get these patients early on in their disease, but we also then need to get them fit enough to tolerate the treatments that we've got, and if we miss out one of those two things, then it doesn't work, we're not getting good survivorship from them. So, I think that was the real driver, that we need to improve the health, the exercise tolerance of this group of people, so that we can give them treatment.’*

Key, Specialist Nurse, Interview, Rosthwaite

Above, Key explains how her service addresses an important barrier in the city where it is based. By providing access to exercise at no (or reduced) cost to patients, services like Rosthwaite aim to make fitness facilities accessible to those who otherwise would be unable to afford them. However, travel to exercise facilities, even if they are free, may limit participation. To address this, three services (Rosthwaite, Greystones, Dalehead) delivered at least some elements in community settings, reducing time and travel demands on patients. However, despite these efforts, some patients remain excluded:

*‘No, I don't think [our service is] fair from a geographical perspective… I don't think we in‐reach enough into those communities. I think if we want people to access exercise classes that may be 30 minutes drive away, then that is going to be a barrier. Likewise, if you haven't got transport it's going to be a barrier. So, I think geographically we've got things that that might inhibit people from joining. Additionally, some of our patients actually come from a different [organisation]… So, for example, we had a referral for a gentleman that's having his diagnostics and treatment within [our organisation], but he lives three hours away and he was like, “I don't know if I want to travel three hours for my prehabilitation assessment…” In terms of digital inclusion, you know, we are going to start to provide digital opportunities for patients… but we know that there's a degree of… barriers… internet connectivity, you know, devices.’*

Mary, Service Lead (Dietitian), Interview, Greystones

Here, Mary acknowledges that, although her service is available at multiple locations, this is not always enough in a semi‐rural location, where some people still need to drive long distances to attend. This exacerbates inequalities because it disadvantages those who reside in rural communities (who may have higher risks of peri‐operative complications [37]). She also expresses discomfort regarding the decision to offer online services, based on digital exclusion, which emphasises how implementing a solution for some patients may diminish accessibility for others.

A further example of challenges in community settings is that resources are often adapted for use in prehabilitation despite being designed for other purposes. For example, leisure centres are designed for leisure, not healthcare. The excerpt below illustrates how the loud music, common to many gym environments, can impair communication during prehabilitation sessions:

*Loud, energetic music playing in the gym space made it particularly difficult to have a conversation… Laurie (exercise practitioner), Kevin (patient) and I found it difficult to follow conversation at a ‘normal’ volume to begin with, unable to hear our own voices properly and each consciously leaning in to speak directly into the others' ears. Kevin, who wore two hearing aids, explained that I should speak directly into his right ear to allow him to hear me more clearly…*

Observation notes taken during a patient's gym session, Rosthwaite

Although the above examples draw on the complexities of community‐based delivery, paradoxes of inequitable access were evident in all services: online delivery (exemplified by Newlands) makes access easy for many, but excludes those without internet access, whereas hospital delivery (Wansfell, Robinson) offers a standardised and appropriate environment, but can result in travel requirements that are unsuitable for some.

### Theme 4 – How services may offer more and/or less than the prototypical prehabilitation model

Across our case study research, we observed that prehabilitation services may simultaneously offer less and more than the prototypical triad of exercise, diet and psychological interventions. They can offer less, in that there are discrepancies between the realities of service delivery and the idealised version of prehabilitation found in the literature. However, they can also offer more, in terms of the benefits that patients perceive, and the teams' aspirations for what it can offer.

One way in which discrepancies between ideals and practice are visible is how the core components of prehabilitation are prioritised in practice. Typically, prehabilitation interventions are represented pictorially as an equal triad of physical fitness, emotional health and nutritional wellness, often depicted in equal measure [32]. However, in practice, certain elements tend to dominate. Across our sample, exercise interventions (often delivered in practical ways, such as exercise classes) took precedence, followed by dietary interventions (typically delivered through advice), then psychological interventions (often implicit):

*‘… the predominant role in the team is to get the patients fitter, really, so from a physical health point of view, it's to optimise them as much as we can, get them as strong as possible, get them cardiovascularly as strong as we can, so when they go into treatment… they're a lot fitter, a lot stronger. And I deal in lung conditions, as well, so if they're going for treatment, it's preparing them for that treatment [from] a physical point of view, so that they can actually cope with the demands of that treatment a lot better.’*

Adam, Physiotherapist, Interview, Greystones

Above, Adam explains the purpose of prehabilitation from his point of view: to make patients ‘strong’ so they can ‘cope’ with treatment. This perspective, articulated by other prehabilitation professionals, was triangulated by the considerable resources dedicated to physical fitness (e.g. staffing, classes, gym sessions). It is notable, however, that exercise‐based interventions take precedence in the discourse around prehabilitation in the medical literature [5], as articulated by Alexander in his discussion of the nuances in the evidence for exercise:

*‘The aerobic is more about what you're trying to get – your heart rate up – and it's got an element of strengthening to it as well. And obviously with exercise it's repeated, you get increasing stimulation [to build] more reserve and build activities… When you look at the evidence base, most of the evidence base in prehab is based in aerobic. Particularly [high intensity interval training] type, you know, high intensity exercise*.

Alexander, Clinical Lead (Anaesthetist/Intensivist), Interview, Rosthwaite

Although the ‘triad’ model of prehabilitation is not implemented equally in practice, our data highlight diverse forms of meaningful participation that fall outside the classical structure. These are rarely captured in service specifications or conventional outcome measures (e.g. 6‐minute walk test; cardiopulmonary exercise testing [3, 4, 5]) and are seldom acknowledged in the academic or patient‐facing literature:

*‘You need to have a plan for the day and you need to have some structure, and I think if you're wallowing around with the shock of a diagnosis – which was very unexpected in my case – and you've got no sort of structure to your day, it can be really unhelpful. So, to be given some sort of plan of eating, plan of exercise, and understanding of the help you can reach out for is very, very helpful… I think they've generated a positivity in me, which has been very useful. And I've felt supported and I've felt that, OK, now I've got all these abilities and knowledge and understanding that I can put into place, and simple exercises and just focusing on my diet and being able to think about the things I may need to put in place is a way of positively accepting each day that there is something that you can always do. So, I think prehab was very good from that point of view: it gave me structure*.

Carolina, Patient (experience of prehabilitation), Interview, Greystones

*‘…this is what I was going to go back to, we do the prehab exercises and it may be that I've just come a little bit more out of my shell, because I'm not‐ I can put on a really good façade and be the life and soul and really in people's faces or I can be comfortable with myself and just sit back and just watch and occasionally say stuff, but with that, we've finished the classes and last week – not yesterday, but last week's class – after the class, we were all, we were actually sitting there, chatting a little bit about our treatments and things, because – I don't know what, I said something, one of the ladies there was going through something and I said, “I didn't realise you'd come through it all, because you look amazing,” and she then started talking about her mouth, how her mouth was still sore, and I was like, “How is that still sore after, like, how long after, because I've got it now?” and she said, “Oh, no, it's for months after,” and all that, and it's like, “Oh, right, this is really informative,” and that little session, it was almost like having a‐ a bit like a counselling session afterwards, but a sharing session, after our gym session, and that was one of the most useful things that has happened since the overall diagnosis, really.’*

Nathan, Patient (experience of prehabilitation), Interview, Newlands

Above, Nathan describes the social benefits of attending a class with others who are facing challenges similar to his own, and Carolina describes how establishing a routine of activities can bring order to an otherwise chaotic time. Although these benefits relate principally to psychosocial wellbeing, we also encountered examples of practical healthcare benefits that go beyond stated service aims:

*‘… just being an advocate for people really, to… give the [general practitioners] a bit of a shove [and] get in touch with them and… formally say as a professional… “this is what we've seen and this is what we think you perhaps need to address”. Whereas the patients… unfortunately these days often have trouble getting appointments, they can't get the point across when they do. So again, just the advocacy role that our service [offers] is really good…*’

Louisa, Occupational Therapist, Interview, Dalehead

Above, Louisa illustrates the benefits of having regular contact with a healthcare professional (described by some as ‘checking in’); it allows health problems to be identified and appropriate referrals made, thereby performing an unofficial screening and advocacy service. This is particularly valuable for patients who may not have the energy or confidence to advocate for themselves, at a time when the NHS appears increasingly fragmented and under unprecedented organisational strain [38, 39, 40].

### Theme 5 – How services use and develop an incomplete evidence base

As would perhaps be expected for a complex, recently developed intervention, the evidence base for prehabilitation remains incomplete [10]. Some cancer types (e.g. colorectal, lung) are better represented than others (e.g. breast, gynaecological, rare cancer types) [10], and some components of prehabilitation are more thoroughly investigated than others (Theme 4). Prehabilitation staff were cognisant of these limitations within the evidence base; in general, services were designed to focus on patient cohorts for whom there was substantial evidence of benefit:

*‘The reason we chose… colorectal and lung is that's where the strongest evidence base is… That's why we chose those pathways…’*

*‘… I was very keen that, when we designed it, that we went where the evidence was because you could have been criticised to say, “well, let's give it to everybody” and we didn't do that because… [you] lose the ability to understand the impact you're making.’*

Alexander, Clinical Lead (Anaesthetist/Intensivist), Interview, Rosthwaite

Here, Alexander recognises that the evidence is ‘strongest’ for particular cancers and explains how his service was designed to align with this. He also outlines risks of going beyond the evidence base in terms of reputational harm and the inability to measure impact. These were recurrently recognised as concerns in the context of prehabilitation, where doubts prevail amongst some colleagues:

*‘Anecdotally from our meetings… the [prehabilitation] consultants are… the ones [who] speak to the… more senior consultants and the other specialities [to] try and promote things. And [they say that] within the anaesthetics department, there's groups [who] are pro‐prehab and can see the value of it, and then there are other groups that say “oh, actually, you know, whoever needs any sort of optimisation for surgery? Let's just get on and do the surgery!” And then… some… consultant surgeons [say] “there's not any… evidence to show it's successful… Why would we refer anyone to you…? …can you show us your impact, your positive impact yet?” And I think that that currently we don't have the data yet – not because it's not there, but… we haven't tried to get it together and do anything with it…’*

Hazel, Peri‐operative Physician, Interview, Newlands

As articulated by Hazel, prehabilitation services often generate new evidence, which extends beyond the quality assurance endeavours that would be encountered in any clinical service; prehabilitation services are challenged not only to maintain high‐quality care, but to justify their existence through showing impact. This indicates that prehabilitation is seen by some as non‐essential, which constitutes an existential threat at a time of financial restraint. Hazel's account also appears to ascribe a generational and professional aspect to this scepticism, hinting that ‘more senior’ consultants may be less likely to endorse this (relatively new) approach.

Despite the incentives to remain within the bounds of evidence, it was also recognised that this may lead to exclusion. While limiting inclusion criteria would be an uncontroversial approach if there was evidence of no benefit, the incomplete nature of the evidence base (which to date has focused on more prevalent cancers and more invasive surgery) means that there are situations where there is no evidence to guide practice. Filling gaps in knowledge was recognised as an important role for services as they become more established:

*‘… for the other tumour groups, particularly skin and upper [gastrointestinal], they've been the last two that have joined [the prehabilitation programme]: they've actually approached us and asked if they could have a prehabilitation offer, and they acknowledge that they want to start small and targeted, to really… focus on measuring success in those tumour groups, because nationally, there isn't [sic] a lot of data around using prehab perhaps with some of these tumour groups…’*

Harper, Macmillan Nurse, Interview, Dalehead

Many prehabilitation staff were involved in the generation of data, comprising both research, and evaluations of their individual services:

*'So I [ran] a randomised trial which demonstrated that exercise intervention before surgery… improved quality of life, and I'm very aware [that], having done an [doctorate] in it, that other research studies have demonstrated that there's improved survival [in] colorectal cancer surgery and since we've introduced our service, we've cut the length of stay for colorectal cancer surgery operations by two days…'*

Peter, Surgeon, Interview, Newlands

Peter describes outcome measures which are commonly used in prehabilitation: quality of life; survival; and duration of hospital stay. Of these, duration of stay was seen as being particularly significant amongst locally generated data, because it speaks to the challenges faced by healthcare organisations, and can be used to inform financial cost–benefit arguments for prehabilitation:

*Interviewer:*
*And are you aware of any impact that the service has had on local patient care or patient outcomes?*

*Arden:*
*‘Definitely on patient outcomes. So, our project manager and admin are really, really good at collecting the outcome measures, and somehow have managed to, in some kind of magical equation, let's call it, have managed to equate that to bed savings. It's not something that… I focus on a day‐to‐day, but we really do have to be aware that this is what is going to get us funding, and if we can prove that we are cost‐effective and efficient, then this is where our funding's going to come from.’*

Arden, Physiotherapist, Interview, Dalehead

Beneath this narrative of ‘proving your worth’ lies an underlying fragility that underscores the precarious nature of services. This places demands on professionals, influencing how care is prioritised and delivered and, as articulated above, how the value of a service can be weighed in different ways, including the need to show value for money in financially challenged NHS organisations [40].

### Theme 6 – How services work with limited resources to provide the most benefit

Professionals working within prehabilitation services recognised the importance of delivering high‐quality, equitable, person‐centred care, while also needing to operate within resource constraints that shape how services are delivered. Idealised versions of care were often challenged by the realities of practice. For example, across our data, the importance of delivering care in person became evident. However, the reality of delivering care in‐person consistently and effectively is thwarted by resource constraints for both prehabilitation services and patients. Below, Zoe underscores the importance of in‐person contact, which enables her to draw on tacit knowledge, body language and non‐verbal cues that may be hard to discern in remote consultations:

*‘We prefer to see patients face to face, because you do get… a better sense and a… better picture from the patient when they're sat in front of you. So, we would always offer that as… a first port of call, really… You can often pick up on more things, certainly from a well‐being point of view; you can pick up on more body language cues and how engaged the patient is or, you know, if they're talking about things, if they're on the verge of tears… which is more difficult to do on the phone because [I] think a lot more of that can be masked a little bit more.’*

Zoe, Occupational Therapist, Interview, Wansfell

It became evident across our observational data how in‐person care enables a more relational and responsive approach. In the below instance, the reflective notes show how in‐person care translates static information (e.g. diagrams) into effective, embodied practice, in those exact moments:

*Elements of the observation served as a reminder that diagrams and written explanations provided to patients are subjective and may not always include enough detail to allow… patient[s] to perform… exercise[s] safely and to the best of their ability. Both therapy assistants acted as a ‘bridge’ in this respect, by monitoring patient posture (e.g. ‘Look up Jim’), [adjusting] positioning and providing advice on how they can improve their performance.’*

Reflective notes from an in‐person exercise class, Greystones

Nevertheless, it was evident that prehabilitation teams recognised the burden that face‐to‐face appointments place on patients (Theme 3). For example, at Wansfell, the team explained that while prehabilitation is available to patients across the citywide catchment area of their organisation, physical clinical space is only available at one hospital, presenting challenges which are further compounded by other organisational constraints:

*‘…we have highlighted that ideally, we would be able to do clinics on different sites. But [estates] is a big issue, and a new hospital was built in City 1, and it was designed a long time before we moved in. So, there weren't a lot of clinic spaces, and so there's not a lot of space. We can't just rock up somewhere and be in clinics, so that feels like something that is a real challenge for us and [it]… doesn't prioritise patients’ time, but I feel quite limited in what I can do to change that.’*

Freya, Service Lead (Physiotherapist), Interview, Wansfell

Within the NHS, prehabilitation is still a relatively new concept, with even the most established services only 10–15 years old. These services must integrate within the existing NHS infrastructure and real estate, nearly half of which is over 40 years old [41], where space is limited and where multiple services co‐exist. Consequently, it becomes essential for the service to develop ways that improve the operational efficiency of the resources available. The value of the support that prehabilitation provides can also depend on individual patients' needs and on prehabilitation professionals' capacity to support them. This is not solely a matter of professional expertise, but also of how care is delivered in practice. In the excerpt below, Joanne divides her attention between two patients with differing needs, impacting the quality of support that each patient receives:

*Over the course of the gym induction, Joanne (exercise specialist) divided her time and attention between… two patients. Each patient had different physical abilities and needs, and they were both using different exercise equipment, guided by Joanne, to suit their physical ability. Consequently, Joanne was moving back and forth between the two patients using different equipment throughout the hour‐long session…*

*Potentially because of spending little time with Carson [one of the two patients] at any given moment, when he was shown a new piece of equipment, there was no explanation regarding (A) what performing the exercise would do for Carson, or (B) how to configure the settings on the machine. There is a difference between ‘knowing the setting’ that the piece of equipment should be set to (this is noted on the patient's personal programme) and knowing how to configure the settings on the piece of equipment itself*.

Reflective notes from a gym induction session, Rosthwaite

Regardless of how prehabilitation was delivered across our case studies, whether in‐person, virtually, in a domiciliary setting or through a combination of these, services were working within resource constraints. In these conditions, implementation becomes a balancing act between maintaining the quality and relational aspect of care and ensuring equitable access to services. Recognising this balance helps to explain how variations in care emerge less from professional shortcomings and more from the systematic constraints influencing care delivery.

## Discussion

Based on data from ‘real‐world’ settings, our work identifies that prehabilitation is multifaceted, carrying different meanings for patients and professionals. Many patients describe and experience prehabilitation in practical ways, focusing on physical preparation for surgery. For many professionals, this extends further, framing prehabilitation not only as a means to improve surgical outcomes, but also to equip patients with tools and knowledge to manage their health during rehabilitation and beyond. In addition to its pragmatic value, prehabilitation provides a sense of continuity for patients [17], offering stability, peer interactions and a familiar healthcare presence. These co‐benefits are not always explicit in service aims and align with the aspirations of healthcare professionals to bring different elements of the ‘pre‐operative puzzle’ together holistically. Nevertheless, engagement with prehabilitation is challenging for many patients and impossible for some. Socio‐economic disparities, regional funding differences and varying service models can create barriers to equitable prehabilitation services [10]. Furthermore, some health professionals remain sceptical about its benefits, meaning that services must constantly prove their worth. Similarly, some patients are hesitant about the value of prehabilitation, particularly when the benefits are unclear and when integration with other aspects of care is lacking.

While prehabilitation represents a superficially simple concept, our research shows how it is a complex intervention [42] that must work with other complex interventions (e.g. surgery, chemotherapy and radiotherapy) at a difficult time in patients' lives. This presents challenges for both patients and services: we show how it competes for patients' time, much of which is already taken up by existing commitments and responsibilities, as well as other healthcare appointments. In some cases, participation can depend on family support, adding a further layer of complexity (Theme 1). This is situated within a broader systemic context that influences the extent to which patients can engage with prehabilitation. At the same time, services operate under precarious conditions with perennial resource constraints (Theme 6). This shapes how care is delivered in practice, including decisions to focus the scope of services, limit availability and restrict locations and opt for particular modes of delivery. This inevitably means that services will suit some better than others, generating the ongoing challenge of balancing feasibility, quality and equity.

Our research shows how some inequalities in service design emerge through different forms of evidence (Theme 5). For instance, at a strategic level, inequalities emerge from research priorities (e.g. related to the amount of research resources focused on certain cancer types), which filter down to influence the evidence base and, in turn, eligibility for prehabilitation (Theme 5). By following an evidence‐based decision‐making approach, services may avoid controversy but unintentionally reproduce research inequalities based on who is represented in the existing literature. Nevertheless, as services evolve and gain social capital, they have the potential to address these inequalities and support other care teams to build an evidence base to better support their patients.

At an operational level, we found that institutional priorities and limitations (e.g. efficiency, financial savings, available space) shape the care patients receive (Theme 6). This is evident in the way that physical fitness interventions are prioritised over other aspects and how prehabilitation teams choose to prove their worth (e.g. reduced duration of stay). This highlights how the value of a service is defined institutionally by healthcare systems and external funders, which may not fully capture other forms of value. However, we show how prehabilitation services are valued by patients and professionals in ways that were not anticipated in service design (Themes 2 and 4). We also explore how prehabilitation can address inequalities but can also widen them based on who can participate; an example of ‘intervention‐generated inequalities’ (Themes 2 and 3) [34]. Our research reinforces the importance of the relational, supportive and person‐centred aspects of prehabilitation, which are distinct from the ‘psychological’ interventions adopted in service specification. Capturing these benefits, perhaps by integrating qualitative approaches into routine service evaluation, would help services to understand their contribution to patient care on a more holistic basis.

While this study takes an innovative approach and expands the current evidence base, it is not without limitations. We restricted our inclusion criteria to services that form a part of NHS treatment pathways, and we were unable to recruit services from Northern Ireland and Scotland. Because case studies are situated in specific settings and circumstances, we would advise caution in directly applying our findings in diverse healthcare systems, but we suggest that our findings provide a useful lens through which to view the problem of health inequality and prehabilitation more generally. Another potential limitation is the reliance on prehabilitation staff as ‘gatekeepers’, which raises the possibility of an element of selectiveness in terms of what we, as visiting researchers, were able to access. Nevertheless, we believe this to be the most comprehensive study of its type, and our analytical decision to focus on common themes across case study sites, the involvement of a diverse research team representing both clinical and academic perspectives, and our reflexive approach to data collection and analysis supports the utility of our findings. We did not conduct any formal analysis of patient information documents as part of the PARITY study, but we identified that this was important during our fieldwork and members of our team investigated it in an associated study [43].

National guidance for prehabilitation before cancer treatment was first issued in the UK in 2019 by Macmillan Cancer Support in partnership with the National Institute for Health Research and the Royal College of Anaesthetists [32]. At the time of its publication, Giles and Cummins raised concerns about the potential of prehabilitation to widen inequalities [44]. Our study validates these concerns to some extent but also shows how prehabilitation can tackle inequalities. For example, by providing supported exercise to people who would otherwise be unable to engage with it (Theme 3), and by providing regular contact with health professionals who can act as advocates during patients' cancer journeys (Theme 4).

Macmillan Cancer Support recently launched updated Prehabilitation for Cancer: Clinical and Implementation Guidelines [45], which explicitly recognises the importance of addressing health inequalities in the design and delivery of prehabilitation services. One limitation of these guidelines is that they do not specify the impact of these inequalities or what services can do to address them. Our research helps to fill this gap by identifying specific examples of how the interaction between health inequalities, service design and service implementation may limit participation in prehabilitation and what services do to address challenges. Examples are included in our results section above and a prioritised list of considerations, which draws on the findings of all stages of the PARITY study [10, 17], is published elsewhere [46]. Prehabilitation before cancer surgery can help address health inequalities by reducing the peri‐operative risk amongst the most vulnerable patients. We summarise the approaches for achieving this aim, which are grounded in our analysis, in Table 3.

**Table 3:** Summary recommendations for helping to address health inequalities through prehabilitation, with links to analytical themes.

Thematic link	Recommendation
Theme 1	Prehabilitation programmes that are supported by, and integrated with, existing systems (e.g. surgery, oncology, peri‐operative care).
Themes 1 and 2	Authentic and personalised interactions with patients which promote engagement with prehabilitation on their own terms.
Theme 3	Attention paid to how services (and the organisations that fund and host them) can help to overcome structural inequalities that restrict access for some patients [46].
Themes 4 and 5	Methods for services to measure their success in the ways that matter most to patients, including social contact; peer support; regular ‘checking in’; and access to informal advocacy.

All prehabilitation services that participated in this study displayed an awareness of the risk of inequitable delivery, but their approaches to mitigation were varied. For example, while some opted for a community‐based approach to address challenges with travel and transport, others opted for an online delivery model. Though influenced in part by the resources available, there was also evidence of tailoring to the systematic challenges prevalent in their catchment areas (e.g. local transport networks and the individual needs of patients). Considering the regional diversity and disparities in the UK, a ‘one‐size‐fits‐all’ approach to delivery is unlikely to be appropriate and prehabilitation services should instead consider how to meet the needs of their population. Considering the uncertainties in the evidence base for prehabilitation, this is likely to rely on the generation of new knowledge, particularly regarding more diverse populations and under‐investigated cancer types.

The PARITY study advances the literature by enhancing the understanding of the interaction between health inequalities and prehabilitation before cancer surgery, and explores the approaches taken by services to address these. While this research is situated specifically within the UK context, previous research has drawn attention to inequalities in prehabilitation in other international contexts [11]. Therefore, while these findings may have relevance beyond the UK, they should be interpreted considering the local context, as is the case with different parts of the UK, where variations in funding, service configurations, local population characteristics and transport infrastructure can shape how inequalities and inequities in prehabilitation are experienced. Future research should build on our findings to design and develop more equitable services and evaluate their impacts in practice.

## Supporting information


**Appendix S1.** Topic guide for orientation interview.

**Appendix S2.** Topic guides for patient, carer and healthcare professional interviews.

**Appendix S3.** Observation guide.

**Figure S1.** Addressing inequities in the design and delivery of prehabilitation in the UK.

**Table S1.** Participant demographic details.

## References

[1] Silver JK . Prehabilitation may help mitigate an increase in COVID‐19 peripandemic surgical morbidity and mortality. Am J Phys Med Rehabil 2020; 99: 459–463. 10.1097/PHM.0000000000001452. PMC7253050 32324618

[2] Molenaar CJ , van Rooijen SJ , Fokkenrood HJ , Roumen RM , Janssen L , Slooter GD . Prehabilitation versus no prehabilitation to improve functional capacity, reduce postoperative complications and improve quality of life in colorectal cancer surgery. Cochrane Database Syst Rev 2022; 5: CD013259. 10.1002/14651858.CD013259.pub2. PMC9118366 35588252

[3] Rosero ID , Ramírez‐Vélez R , Lucia A , et al. Systematic review and meta‐analysis of randomized, controlled trials on preoperative physical exercise interventions in patients with non‐small‐cell lung cancer. Cancers (Basel) 2019; 11: 944. 10.3390/cancers11070944. PMC6678369 31284372

[4] Lambert JA , Hayes LD , Keegan TJ , Subar DA , Gaffney CJ . The impact of prehabilitation on patient outcomes in hepatobiliary, colorectal, and upper gastrointestinal cancer surgery: a PRISMA‐accordant meta‐analysis. Ann Surg 2021; 274: 70–77. 10.1097/SLA.0000000000004527. 33201129

[5] Waterland JL , McCourt O , Edbrooks L , et al. Efficacy of prehabilitation including exercise on postoperative outcomes following abdominal cancer surgery: a systematic review and meta‐analysis. Front Surg 2021; 8: 628848. 10.3389/fsurg.2021.628848. PMC8017317 33816546

[6] Bundred JR , Kamarajah SK , Hammond JS , Wilson CH , Prentis J , Pandanaboyana S . Prehabilitation prior to surgery for pancreatic cancer: a systematic review. Pancreatology 2020; 20: 1243–1250. 10.1016/j.pan.2020.07.411. 32826168

[7] Stewart H , Zhang X , Hirst L , et al. Prehabilitation before cancer surgery in the UK National Health Service: what services exist, and how do they address health inequalities? PLoS One 2026; 21: e0336005. 10.1371/journal.pone.0336005. PMC13152163 42096417

[8] Scottish Government . Cancer Prehabilitation in Scotland: Report on Survey Findings. 2023. https://www.gov.scot/binaries/content/documents/govscot/publications/research‐and‐analysis/2023/04/cancer‐prehabilitation‐scotland‐report‐survey‐findings/documents/cancer‐prehabilitation‐scotland‐report‐survey‐findings/cancer‐prehabilitation‐scotland‐report‐survey‐findings/govscot%3Adocument/cancer‐prehabilitation‐scotland‐report‐survey‐findings.pdf (accessed 16/06/2026).

[9] Pufulete M , Coyle V , Provan D , et al. Prehabilitation provision and practice in the UK: a freedom of information survey. Br J Anaesth 2024; 132: 815–819. 10.1016/j.bja.2024.01.013. 38326209

[10] Stewart H , Stanley S , Zhang X , et al. The inequalities and challenges of prehabilitation before cancer surgery: a narrative review. Anaesthesia 2025; 80(Suppl 2): 75–84. 10.1111/anae.16502. PMC11744418 39775660

[11] Lee D , Wang A , Augustin B , Buajitti E , Tahasildar B , Carli F , Gillis C . Socioeconomic status influences participation in cancer prehabilitation and preparation for surgical recovery: a pooled retrospective analysis using a validated area‐level socioeconomic status metric. Eur J Surg Oncol 2023; 49: 512–520. 10.1016/j.ejso.2022.10.023. 36435646

[12] Barnes K , Hladkowicz E , Dorrance K , et al. Barriers and facilitators to participation in exercise prehabilitation before cancer surgery for older adults with frailty: a qualitative study. BMC Geriatr 2023; 23: 356. 10.1186/s12877-023-03990-3. PMC10242997 37280523

[13] Kovoor JG , Nann SD , Chambers C , et al. Prehabilitation before general surgery: worth the effort? J Perioper Pract 2024; 34: 219–225. 10.1177/17504589231214395. 38149496

[14] Marmot M . Health equity in England: the Marmot review 10 years on. BMJ 2020; 368: m693. 10.1136/bmj.m693. 32094110

[15] Zolfaghari EJ , Ayoade O , Antonoff MB . Barriers to fitness: disparities in preoperative exercise access for patients with lung cancer. J Thorac Cardiovasc Surg 2025; 169: e39. 10.1016/j.jtcvs.2024.11.023. 39674938

[16] NHS England . The NHS Constitution for England. 2023. https://www.gov.uk/government/publications/the‐nhs‐constitution‐for‐england/the‐nhs‐constitution‐for‐england (accessed 16/06/2026).

[17] Wareing L , Hirst Y , Shelton C , et al. Identifying quality and inequality in prehabilitation services before cancer surgery: a Delphi study informed by lived and professional experience. Eur J Cancer Care (Engl) 2025; 2025: 9308284. 10.1155/ecc/9308284.

[18] Crowe S , Cresswell K , Robertson A , Huby G , Avery A , Sheikh A . The case study approach. BMC Med Res Methodol 2011; 11: 100. 10.1186/1471-2288-11-100. PMC3141799 21707982

[19] Guba EG , Lincoln YS . Naturalistic Inquiry. Beverly Hills, CA: Sage Publications, 1985.

[20] Shelton CL , Mort MM , Smith AF . ‘It's learned on the job and it depends who you're with.’ An observational qualitative study of how internal jugular cannulation is taught and learned. J Intensive Care Soc 2018; 19: 26–34. 10.1177/1751143717728631. PMC5810876 29456598

[21] Shelton CL . In Search of the ‘Good Anaesthetic’ for Hip Fracture Repair: Difference, Uncertainty and Ideology in an Age of Evidence‐Based Medicine (PhD thesis). 2019. https://eprints.lancs.ac.uk/id/eprint/137973/1/2019SheltonPhD.pdf (accessed 16/06/2026).

[22] Malterud K , Siersma VD , Guassora AD . Sample size in qualitative interview studies: guided by information Power. Qual Health Res 2016; 26: 1753–1760. 10.1177/1049732315617444. 26613970

[23] National Institute for Health and Care Research . Mapping and identifying quality and inequality in prehabilitation for cancer surgery: evidence for improvement. 2023. https://fundingawards.nihr.ac.uk/award/NIHR134282 (accessed 30/01/2026).

[24] Department for Constitutional Affairs . Mental Capacity Act 2005 Code of Practice. 2007. https://assets.publishing.service.gov.uk/media/5f6cc6138fa8f541f6763295/Mental‐capacity‐act‐code‐of‐practice.pdf (accessed 16/06/2026).

[25] Rycroft‐Malone J , Kitson A , Harvey G , McCormack B , Seers K , Titchen A , Estabrooks C . Ingredients for change: revisiting a conceptual framework. Qual Saf Health Care 2002; 11: 174–180. 10.1136/qhc.11.2.174. PMC1743587 12448812

[26] Kitson AL , Rycroft‐Malone J , Harvey G , McCormack B , Seers K , Titchen A . Evaluating the successful implementation of evidence into practice using the PARiHS framework: theoretical and practical challenges. Implement Sci 2008; 3: 1. 10.1186/1748-5908-3-1. PMC2235887 18179688

[27] Braun V , Clarke V . *U*sing thematic analysis in psychology. Qual Res Psychol 2006; 3: 77–101. 10.1191/1478088706qp063oa.

[28] Shelton CL , Smith AF , Mort M . Opening up the black box: an introduction to qualitative research methods in anaesthesia. Anaesthesia 2014; 69: 270–280. 10.1111/anae.12517. 24548356

[29] O'Brien BC , Harris IB , Beckman TJ , Reed DA , Cook DA . Standards for reporting qualitative research: a synthesis of recommendations. Acad Med 2014; 89: 1245–1251. 10.1097/ACM.0000000000000388. 24979285

[30] Grimmett C , Bradbury K , Dalton SO , Fecher‐Jones I , Hoedjes M , Varkonyi‐Sepp J , Short CE . The role of behavioral science in personalized multimodal prehabilitation in cancer. Front Psychol 2021; 12: 634223. 10.3389/fpsyg.2021.634223. PMC7921482 33664701

[31] Watson A , Murnen SK , College K . Gender differences in responses to thin, athletic, and hyper‐muscular idealized bodies. Body Image 2019; 30: 1–9. 10.1016/j.bodyim.2019.03.010. 31071678

[32] Macmillan Cancer Support . Prehabilitation for people with cancer: principles and guidance for prehabilitation within the management and support of people with cancer. 2019. www.macmillan.org.uk/assets/prehabilitation‐guidance‐for‐people‐with‐cancer.pdf (accessed 16/06/2026).

[33] Ligibel JA , Bohlke K , May AM , et al. Exercise, diet, and weight management during cancer treatment: ASCO guideline. J Clin Oncol 2022; 40: 2491–2507. 10.1200/JCO.22.00687. 35576506

[34] Lorenc T , Petticrew M , Welch V , Tugwell P . What types of interventions generate inequalities? Evidence from systematic reviews. J Epidemiol Community Health 2013; 67: 190–193. 10.1136/jech-2012-201257. 22875078

[35] Wan YI , McGuckin D , Fowler AJ , Prowle JR , Pearse RM , Moonesinghe SR . Socioeconomic deprivation and long‐term outcomes after elective surgery: analysis of prospective data from two observational studies. Br J Anaesth 2021; 126: 642–651. 10.1016/j.bja.2020.10.019. 33220938

[36] Chauhan A , Walton M , Manias E , et al. The safety of health care for ethnic minority patients: a systematic review. Int J Equity Health 2020; 19: 118. 10.1186/s12939-020-01223-2. PMC7346414 32641040

[37] Annibal I . Rural Health Inequalities: The Challenges Facing England's Countryside and Coasts. 2024. https://nhsproviders.org/resources/rural‐health‐inequalities‐the‐challenges‐facing‐englands‐countryside‐and‐coasts (accessed 16/06/2026).

[38] Shepherd S . Coordinated care: a patient perspective on the impact of a fragmented system of care on experiences and outcomes, drawing on practical examples. Future Hosp J 2016; 3: 136–138. 10.7861/futurehosp.3-2-136. PMC6465825 31098207

[39] Care Quality Commission . The state of health care and adult social care in England 2024/25. 2025. https://www.cqc.org.uk/sites/default/files/2025‐10/20251024_stateofcare2425_print.pdf (accessed 16/06/2026).

[40] Jeffries D , Wickens C . Tight Budgets and Tough Choices: The Reality of an NHS Living Within Its Financial Means. 2025. https://www.kingsfund.org.uk/insight‐and‐analysis/long‐reads/tight‐budgets‐tough‐choices (accessed 16/06/2026).

[41] Kirk‐Wade E . Hospital building in England: Plans and progress. 2024. https://commonslibrary.parliament.uk/hospital‐building‐in‐england‐plans‐and‐progress/ (accessed 16/06/2026).

[42] Skivington K , Matthews L , Simpson SA , et al. A new framework for developing and evaluating complex interventions: update of Medical Research Council guidance. BMJ 2021; 374: n2061. 10.1136/bmj.n2061. PMC8482308 34593508

[43] Stanley S , Stewart H , Shelton C . Promoting or pressurising participation? A discourse analysis of online patient information resources about prehabilitation before cancer treatment. PLoS One 2025; 20: e0339169. 10.1371/journal.pone.0339169. PMC12725656 41433282

[44] Giles C , Cummins S . Prehabilitation before cancer treatment. BMJ 2019; 366: l5120. 10.1136/bmj.l5120. 31413000

[45] Macmillan Cancer Support . Prehabilitation for People with Cancer: Clinical and Implementation Guidelines. 2025. https://www.macmillan.org.uk/dfsmedia/1a6f23537f7f4519bb0cf14c45b2a629/22869‐10061/prehabilitation‐for‐people‐with‐cancer‐clinical‐and‐implementation‐guidelines (accessed 16/06/2026).

[46] Zhang X , Hadley L , Hadley C , et al. Improving equity in prehabilitation before cancer surgery: consensus‐based considerations for leaders and practitioners: a nominal group technique study. Anaesth Rep 2026; 14: e70085. 10.1002/anr3.70085. PMC13369003 42459925

