# Supplementary material for: Addressing inequities in the design and delivery of prehabilitation in the UK: case studies of challenges, complexities and good practice*
Source: Anaesthesia. 2026 Jul 20;81(11):1510–25. doi: 10.1111/anae.70286 (PMC13659788; doi:10.1111/anae.70286)
Supplement: Supplementary file 1 [file ANAE-81-1510-s001.pdf]

PARITY
Prehabiliation for Cancer Surgery: Quality and Inequality

Lancaster University

PARITY CASE STUDY Semi-structured PATIENT INTERVIEW SCHEDULE _Version 1.0 Date 31/03/2023

This is a semi-structured interview. The interviewer will use these as prompts as part of the interview to guide the interviewee to capture in depth information about Prehabilitation. The questions may be rephrased or new questions may be added to explore further.

Introduction:

Thank them for giving up their time to speak with you (make them feel welcomed and valued).
Explain how the interview will work.

Cover:

- Length and format
- How it will be recorded
- Reassure them that you can repeat or clarify questions
- Reassure them there are no right or wrong answers
- You can take a break or stop at any time
- Allow for any questions before you provide a brief overview of your research
- Confirm consent verbally before starting the interview

Brief Personal Background and diagnosis

1) To begin with, please can you tell me a little bit about yourself, for instance about your day-to-day life, profession, how you like spending your days, hobbies, family and support environment .
2) How would you describe your health and how you cared for your health in general before your diagnosis?
3) Thank you so much, and before we continue talking about the aims of this study, please could you describe your cancer diagnosis in your own words and your experience up to date.
   Prompts:
   - Would you mind telling me/us about its impact on your day-to-day life?
   - How it might have affected your finances.
   - Things you were able to do before and now might be challenging
4) As part of our research question, we are also interested to find out who you think is part of your cancer care team since you received your diagnosis.
   a. Why?

Thank the participant before moving to the next stage.

About Prehab

I'm going to read you a standardised paragraph about Prehabilitation to remind you the aim of the study. We are interested in improving Prehabilitation services before cancer surgery. Prehabilitation describes a number of interventions that aim to improve patients' physical, nutritional and psychological wellbeing before receiving surgery. This is designed to help individuals to be better

prepared physically and emotionally for major events such as cancer surgery, with an aim to improve their outcomes.

1) What are your immediate thoughts and feelings about what I have just explained about prehabilitation?
    a. Prompts: awareness and knowledge

2) Have you been offered any <u>activities, support, tools</u> that fit the description of Prehabilitation before your operation? **(go to Q4 if they say no)**

(e.g. Surgery school, fit4surgery, exercise classes etc)

Can you please describe in detail what these were

Prompts if they were offered prehab for each type:

- When was it first offered?
- Why was it offered?
- Who offered it?
- What was offered?
- What was not offered?
- How did they decide to do it? Was it a shared decision?
- Were they able to contribute to what they were asked to do depending on the type of activity?
- Was it tailored to their needs?
- What were the challenges of attending appointments/completing task?
- How were they informed about what to do?
- What were the tools/resources shared by the healthcare professionals? Anything for family members?
- Any ideas about the knowledge and awareness about the patient's circumstances and Prehabilitation plan between people in their cancer care team

3) If they were offered multiple prehab options, how are these connected to each other? Was that explained?
4) <u>If they were not offered Prehab,</u> explore if they were offered any of the following:
    a. Leaflets/websites
    b. Specific charity guidance
    c. Community-based activities (self-referral to physio, gym)
    d. Self-referral
    e. Primary care-based activities.
    f. Self-care activities (continue exercising as usual, riding a bike etc. or change in behaviours .e.g. reduced activities

5) How did they receive information about these different services?
6) What did they take up?
7) Who delivered it?

8) Why?
9) How?
10) What was involved?

Additional questions for the preoperative period (tailor the following questions depending on the proximity to surgery)

11) Did you do anything different to prepare yourself for surgery that we have not discussed so far?
12) What did you find meaningful to do while waiting for your surgery?
13) How do you think all these activities impacted your health?
    a. Any feedback from their cancer care team? What was it?
14) How do you think the activities impacted day-to-day living?
    a. Access / Transportation/Affordability/understanding (explore inequalities)
       Prompts for exploring inequalities: Do they think the activities were affected by any of the following:
       Money and resources,
       Work, unemployment or education,
       Where they live and their housing,
       Limited transport,
       Access to the internet,
       Communication barriers,
       Barriers due to protected characteristics or vulnerabilities

    b. Impact on Family and Friends – were they provided with anything to help them?
15) What would they want to happen differently to make it better?
16) Is there anything else they would like to add?

Thank the participant for their contribution, remind them that you are going to stop recording, and remind how they can contact you if they have any questions.

Parity Case Study Semi-Structured Carer Interview Schedule _Version 1.0 Date 31/03/2023
This is a semi-structured interview. The interviewer will use these as prompts as part of the interview to guide the interviewee to capture in depth information about Prehabilitation. The questions may be rephrased or new questions may be added to explore further.

Introduction:
Thank them for giving up their time to speak with you (make them feel welcomed and valued).
Explain how the interview will work.

Cover:

- Length and format
- How it will be recorded
- Reassure them that you can repeat or clarify questions
- Reassure them there are no right or wrong answers
- You can take a break or stop at any time
- Allow for any questions before you provide a brief overview of your research
- Confirm consent verbally before starting the interview

Brief Personal Background and diagnosis

1) To begin with, please can you tell me a little bit about yourself and the patient you are caring for. For instance about your day-to-day life, profession, how you like spending your days, hobbies, family and support environment .
2) How would you describe [PaTIENTS name] health and how you cared for your health in general before diagnosis?
3) Thank you so much, and before we continue talking about the aims of this study, please could you describe their cancer diagnosis in your own words and your experience up to date.
   Prompts:
   - Would you mind telling me/us about its impact on day-to-day life?
   - How it might have affected their finances.
   - Things you were able to do before and now might be challenging

4) As part of our research question, we are also interested to find out who you think is part of [PaTIENTS name] cancer care team since [PaTIENTS name] received cancer diagnosis.
   a. Why?

Thank the participant before moving to the next stage.

About Prehab

I'm going to read you a standardised paragraph about Prehabilitation to remind you the aim of the study. We are interested in improving Prehabilitation services before cancer surgery. Prehabilitation describes a number of interventions that aim to improve patients' physical, nutritional and psychological wellbeing before receiving surgery. This is designed to help individuals to be better

prepared physically and emotionally for major events such as cancer surgery, with an aim to improve their outcomes.

1) What are your immediate thoughts and feelings about what I have just explained about prehabilitation?
    a. Prompts: awareness and knowledge

2) Have you been offered any activities that fit the description of Prehabilitation before [PaTIENTS name] operation? **(go to Q4 if they say no)**

(e.g. Surgery school, fit4surgery, exercise classes etc)

Can you please describe in detail what these activities were

Prompts if they were offered prehab for each type:

- When was it first offered?
- Why was it offered?
- Who offered it?
- What was offered?
- What was not offered?
- How did they decide to do it? Was it a shared decision?
- Were they able to contribute to what they were asked to do depending on the type of activity?
- Was it tailored to their needs?
- What were the challenges of attending appointments/completing task?
- How were they informed about what to do?
- What were the tools/resources shared by the healthcare professionals? Anything for family members?
- Any ideas about the knowledge and awareness about the patient's circumstances and Prehabilitation plan between people in their cancer care team

3) If they were offered multiple prehab options, how are these connected to each other? Was that explained?
4) <u>If the patient was not offered Prehab,</u> explore if they were offered any of the following:
    a. Leaflets/websites
    b. Specific charity guidance
    c. Community-based activities (self-referral to physio, gym)
    d. Self-referral
    e. Primary care-based activities.
    f. Self-care activities (continue exercising as usual, riding a bike etc. or change in behaviours .e.g. reduced activities

5) How did they receive information about these different services?
6) What did they take up?
7) Who delivered it?
8) Why?

9) How?
10) What was involved?

Additional questions for the preoperative period (tailor the following questions depending on the proximity to surgery)

11) Did you do anything different to prepare [PaTIENTS name] for surgery that we have not discussed so far?
12) What did they find meaningful to do while waiting for the surgery?
13) How do you think all these activities impacted [PaTIENTS name] health?
    a. Any feedback from their cancer care team? What was it?
14) How do you think the activities impacted day-to-day living?
    a. Access / Transportation/Affordability/understanding (explore inequalities)
       Prompts for exploring inequalities: Do they think the activities were affected by any of the following:
       Money and resources,
       Work, unemployment or education,
       Where they live and their housing,
       Limited transport,
       Access to the internet,
       Communication barriers,
       Barriers due to protected characteristics or vulnerabilities

    b. Impact on Family and Friends – were they provided with anything to help them?
15) What would they want to happen differently to make it better?
16) Is there anything else they would like to add?

Thank the participant for their contribution, remind them that you are going to stop recording, and remind how they can contact you if they have any questions.

PARITY
Prehabiliation for Cancer Surgery: Quality and Inequality

Lancaster University

PARITY Case Study _ Healthcare professional interview schedule informed by PARIHS Framework

**Introduction / housekeeping**	Thank you<br>Recording<br>Verbal consent<br>Withdrawal<br>Restate the aims
**About the interviewee**	1) Can you please tell me about yourself, your background and your current role in Prehabilitation before cancer surgery?
**Description of prehab**	2) Would you be able to describe the Prehabilitation services provided at your hospital from your point of view?<br><br>Prompts: what does it look like, what does it involve, what cancer sites/sub-types are included, aims and objectives and future plans.
**Evidence**<br>Research<br>Clinical Experience<br>Patient Experience<br>Information and Data from the local context	3) GENERAL: Would you be able to tell me about how the Prehabilitation services at your hospital were conceived?<br>Prompts:<br>Is there specific evidence/ publication that lead to the design?<br>What were the drivers of implementation?<br>Can you tell me about the rationale and priorities that informed it?<br>How was it funded?<br>Were there specific people whose experience informed practice?<br>How did you and your colleagues decide on the focus of their services?<br><br>4) Did you pilot the current programme before implementation?<br>If yes, what were the learnings from it<br>If not, what was the reason why they did not.<br><br>5) In what ways have you been using patient experience for informing the design of the service?<br>Prompts:<br>a) Different datasets<br>b) What conclusions have they made, if any<br>c) How relevant do they think it is to the service design<br><br>6) What are your thoughts about how well the care provided matches patient preferences and needs?<br><br>7) How do you tailor care for different patients with different needs?<br><br>8) What is the impact of your services on local patient care and outcomes?<br>Prompts:

	a) How do they evaluate the impact?<br>b) How does it relate to local cancer incidence, survival and mortality rates?<br>9) What are the future plans if any to improve what they are already providing and what is the reason/rationale behind it?
**Context**<br>Culture<br>Leadership<br>Evaluation	10) **GENERAL:** Would you be able to tell us about the general organisational culture surrounding Prehabilitation service?<br><br>Prompts:<br>a) Personal growth / initiatives<br>b) Leadership, autonomy<br>c) Deadlines?<br>d) Performance measures<br>e) Expectations<br>f) How they are evaluated as providers?<br>g) Employment / feeling financially secure<br>h) Staff turnover<br><br>11) How about the resources required to deliver Prehabilitation care?<br>a. Do they have sufficient resources? If not, what is needed?
**Facilitation**<br>Purpose<br>Role<br>Skills and Attributes	12) **General:** What are your thoughts in general on the day to day delivery of Prehabilitation provided at your service/ward that would make it successful or not successful?<br>Prompts:<br>a) Are there any barriers? Why? Can you please elaborate?<br>b) Are there any facilitators? Why? Can you please elaborate?<br>c) What would make it easier?<br>d) What is required to make it successful?<br>e) Do you think the service is fair? Why -can you please elaborate? What would make it fair?<br>f) Do you think the service is accessible? Why – can you please elaborate? What would make it accessible?<br>g) Who benefits? why?<br>h) Who does not benefit? Why?<br><br>13) Who are the facilitators? Why? What skills /characteristics do they need to have to deliver equitable and effective Prehabilitation?
**Closing/Debrief**	14) Would you like to add anything else that I missed and that you would like to share about your services?<br><br>Now, I'll turn off the recorder. Thank you for your participation.
