# Supplementary material for: Addressing inequities in the design and delivery of prehabilitation in the UK: case studies of challenges, complexities and good practice*
Source: Anaesthesia. 2026 Jul 20;81(11):1510–25. doi: 10.1111/anae.70286 (PMC13659788; doi:10.1111/anae.70286)
Supplement: Supplementary file 2 [file ANAE-81-1510-s002.pdf]

Appendix S1: Topic guide for orientation interview

Orientation Checklist		
The name of the Case Study Site Hospital		
Lead Contact (name, email, phone)		
R&D Contact (name, email, phone))		
Dates agreed for observations (DD/MM/YYYY)	1.<br>2.<br>3.<br>4.<br>5.	
PARITY Researchers who will carry out observations		
Risk Assessment (e.g. provide details for PPE requirements)		
Cancer sites observed (List all the cancer types of patients who are referred into this service)		
Clinics on the day	Universal interventions<br>Targeted Interventions<br>Specialist Interventions	
Reasonable Adjustment Requirements	Do you need to arrange translators?<br>Do you need easy to read documents?<br>Other	

Prehabilitation Providers<br>(List all the specialties included in the clinics e.g. physiotherapist, dietician, psychologist, other specialties)		

Orientation Interview Schedule

1) Confirm consent and whether the participant is happy to be recorded and transcribed.

2) Ask the respondents to provide enhanced demographics and statistical information based on the mapping questionnaire outcomes using open questioning.
    a. Discuss in detail the funding arrangements for their Prehabilitation service
    b. Discuss in detail how the service is managed (clinical vs non-clinical, single specialty vs multi-disciplinary)
    c. Discuss in detail the governance details (e.g. how its performance and outcomes are measured)
    d. How and where Prehabilitation is provided (specific location at the hospital)
    e. What decision making processes are involved in enrolling patients in the service
3) Ask the key informants/leads for organisational literature connected with their service. (e.g. Clinical guidelines, Service level agreements, patient forms)

This is a semi-structured interview. The interviewer will use these as prompts as part of the orientation interview to guide the interviewee to capture the aforementioned points. The questions may be rephrased or new questions may be added to explore further.

Ask the programme lead if they also wish to take part in the detailed interview as part of the orientation checklist interview
