# Supplementary material for: Addressing inequities in the design and delivery of prehabilitation in the UK: case studies of challenges, complexities and good practice*
Source: Anaesthesia. 2026 Jul 20;81(11):1510–25. doi: 10.1111/anae.70286 (PMC13659788; doi:10.1111/anae.70286)
Supplement: Supplementary file 3 [file ANAE-81-1510-s003.pdf]

PARITY
Prehabiliation for Cancer Surgery: Quality and Inequality

Appendix S3: Observation guide

Lancaster University

PARITY Case Study Observation Data Collection Form Version 1 (02/02/2023)

PARITY Researcher Name:

**Site Name:**	
**Department:**	
**Date of observation:**	
**Time of observation:**	
**The names of the healthcare professionals present:**	
**Number of people on the ward/unit**	
**Sequence of events:**	
**Interruptions:**	
**Visual aids for patients (e.g. TV, leaflets, posters):**	

General mood:	

The purpose of the observation:<br><br>**“Pay attention to inequalities” – e.g.** protected characteristics, barriers to prehabilitation, access.			
**Dimensions based on PARIHS Framework**			
	Low	High	Observations / Interview data
**Evidence**			
Research	Poorly conceived, designed, and/or executed research • Seen as the only type of evidence • Not valued as evidence • Seen as certain	Well-conceived, designed, and executed research, appropriate to the research question<br>• Seen as one part of a decision<br>• Valued as evidence• Lack of certainty acknowledged<br>• Social construction acknowledged<br>• Judged as relevant<br>• Importance weighted • Conclusions drawn	

Clinical Experience	• Anecdotal, with no critical reflection and judgment<br>• Lack of consensus within similar groups • Not valued as evidence<br>• Seen as the only type of evidence	Clinical experience and expertise reflected upon, tested by individuals and groups • Consensus within similar groups<br>• Valued as evidence• Seen as one part of the decision<br>• Judged as relevant	
		• Importance weighted • Conclusions drawn	
Patient Experience	Not valued as evidence<br>• Patients not involved<br>• Seen as the only type of evidence	Valued as evidence • Multiple biographies used<br>• Partnerships with healthcare professionals • Seen as one part of a decision<br>• Judged as relevant<br>• Importance weighted • Conclusions drawn	

Information Data from the local context	Not valued as evidence • Lack of systematic methods for collection and analysis<br>• Not reflected upon<br>• No conclusions drawn	Valued as evidence<br>• Collected and analysed systematically and rigorously<br>• Evaluated and reflected upon<br>• Conclusions drawn	
**Context**			
**Culture**	Unclear values and beliefs<br>• Low regard for individuals<br>• Task-driven organization<br>• Lack of consistency• Resources not allocated<br>• Well integrated with strategic goals	Able to define culture(s) in terms of prevailing values/beliefs<br>• Values individual staff and clients<br>• Promotes learning organisation • Consistency of individual's role/experience to value Relationship with others Teamwork Power and authority Rewards/recognition<br>• Resources—human, financial, equipment – allocated<br>• Initiative fits with strategic goals and is a key practice/patient issue	

Leadership	Traditional, command, and control leadership • Lack of role clarity • Lack of teamwork • Poor organizational structures • Autocratic decision-making processes • Didactic approaches to learning/teaching/managing	Transformational leadership • Role clarity • Effective teamwork • Effective organizational structures • Democratic-inclusive decision-making processes •Enabling/empowering approach to teaching/learning/managing	
Evaluation	Absence of any form of feedback • Narrow use of performance information sources • Evaluations rely on single rather than multiple methods	Feedback on Individual Team System performance • Use of multiple sources of information on performance • Use of multiple methods Clinical Performance Economic Experience evaluations	
**Facilitation**			
Purpose	Task	Holistic	
Role	Doing for others	Enabling others	

	• Episodic contact• Practical/technical help<br>• Didactic, traditional approach to teaching • External agents • Low intensity—extensive coverage	• Sustained partnership • Developmental<br>• Adult learning approach to teaching<br>• Internal/external agents• High intensity—limited coverage	
Skills and Attributes	Task/doing for others • Project management skills<br>• Technical skills<br>• Marketing skills<br>• Subject/technical/clinical credibility	Holistic/enabling others<br>• Cocounselling<br>• Critical reflection<br>• Giving meaning<br>• Flexibility of role<br>• Realness/authenticity	
Researcher Reflections			
How do you feel today?			

PARITY

Prehabiliation for Cancer Surgery: Quality and Inequality

Lancaster University

How do you feel about the observation you carried out?

<u>Free form field notes:</u>
