# Supplementary material for: Addressing inequities in the design and delivery of prehabilitation in the UK: case studies of challenges, complexities and good practice*
Source: Anaesthesia. 2026 Jul 20;81(11):1510–25. doi: 10.1111/anae.70286 (PMC13659788; doi:10.1111/anae.70286)
Supplement: Supplementary file 4 [file ANAE-81-1510-s004.pdf]

Addressing inequities in the design and delivery of prehabilitation in the UK

Case studies of challenges, complexities and good practice

6 Case study sites

Representing a mix of services:

- Remote
- Urban
- In set-up
- Semi-rural
- Recent
- Hospital
- Well-established
- In-person

47 Interviews with professionals

47 Interviews with patients/carers

28 Observations of practice

Inductive thematic analysis by a team of researchers

What was identified

- How patients' circumstances influence their ability to engage.
- How the value of prehabilitation to patients depends on how it is introduced and understood.
- How services can address – but also exacerbate – health inequalities.
- How services may offer more and/or less than the prototypical prehabilitation model.
- How services use and develop an incomplete evidence base.
- How services work with limited resources to provide the most benefit.

To move towards more equitable prehabilitation, we need:

- Programmes that are supported by, and integrated with, existing systems
- Authentic and personalised interactions with patients
- Ways to overcome structural inequalities
- To recognise and value holistic care

C Hadley, X Zhang, H Stewart, et al. Anaesthesia 2026

FUNDED BY NIHR | National Institute for Health and Care Research

Health & Medicine | Lancaster University

PARITY
Prehabilitation for Cancer Surgery: Quality and Inequality
