# Supplementary material for: Addressing inequities in the design and delivery of prehabilitation in the UK: case studies of challenges, complexities and good practice*
Source: Anaesthesia. 2026 Jul 20;81(11):1510–25. doi: 10.1111/anae.70286 (PMC13659788; doi:10.1111/anae.70286)
Supplement: Supplementary file 5 [file ANAE-81-1510-s005.pdf]

**Table S1:** Characteristics of professional and patient participants included in interviews and small group observations. This does not include participants from public or large group observations. *Demographic data were not collected for the 11 carer/relative participants – these are reported for patients only.

| Participant type | Characteristic | Category | Number (proportion) |
|---|---|---|---|
| **Professionals:** | Gender | Male | 7 (14%) |
| | | Female | 43 (86%) |
| | Profession | Physiotherapist | 8 (16 %) |
| | | Doctor | 7 (14 %) |
| | | Occupational Therapist | 6 (12 %) |
| | | Nurse | 6 (12 %) |
| | | Administrator | 3 (6 %) |
| | | Dietitian | 4 (8 %) |
| | | Manager | 3 (6 %) |
| | | Exercise professionals | 3 (6%) |
| | | Speech and Language Therapist | 2 (4 %) |
| | | Other | 8 (16%) |
| **Patients / carers:** | Role | Patient | 61 (85%) |
| | | Relative / Carer | 11 (15%) |
| | Gender* | Male | 20 (33%) |
| | | Female | 36 (59%) |
| | | Not declared / missing | 5 (8%) |
| | Age* | Working age | 28 (45%) |
| | | Retirement age | 22 (36%) |
| | | Not declared / missing | 11 (18%) |
| | Ethnicity* | White | 33 (54%) |
| | | Minority ethnic groups | 5 (8%) |
| | | Not declared / missing | 23 (38%) |
| | Experience of prehabilitation* | Yes | 42 (69%) |
| | | No | 19 (31%) |
